# Proceedings of the Sixteenth Annual Meeting of the American Dental Association

**Published:** 1876-09

**Authors:** 


AMERICAN JOURNAL

OF

### DENTAL SCIENCE.

Vol. X. THIRD SERIES—SEPTEMBER, 1876. No. 5.

### ARTICLE I.

### *The American Dental Association.—Sixteenth Annual Session.*

According to announcement this Association held its annual meeting in Philadelphia, commencing Tuesday morning, August 1st, 1876, at 10 o'clock, in the Chapel of the Arch Street Methodist Episcopal Church, S. E. Corner of Broad and Arch Streets. The following dentists were present at the opening of the first day's session :

Drs. F. Abbott, New York; John Allen, New York; W. H. Allen, New York; J. J. Anderson, Springfield, Ill.; W. H. Atkinson, New York; W. B. Acheson, Ohio; T. L. Buckingham, Philadelphia; Alonzo Boice, Philadelphia; C. M. Bailey, Minneapolis, Minn.; E. A. Bogue, New York; G. T. Barker, Philadelphia; C. H. Biddle, New York; A. H. Brockway, New York; George H. Cushing, Chicago; J. W. Clowes, New York; C. D. Cook, New York; J. N. Crouse, Chicago; M. S. Dean, Chicago; M. W. Foster, Baltimore; George L. Field; Detroit, J. Foster

Flagg, Philadelphia; F. J. S. Gorgas, Baltimore; E. S. Gaylord, New Haven, Conn.; M. H. Goddard, Louisville, Ky.; J. Hooper, Covington, Ky.; A. W. Harlan, Chicago; J. L. Hill, Gettysburg, Pa.; R. F. Hunt, Washington, D. C.; F. A. Hunter, Cincinnati; C. Kingsbury, Philadel, phia; G. W. Klump, Williamsport, Pa.; M. Lukens Long-Philadelphia; A. E. Lyman, Warren, Ohio; G. B. McDonneld, Erie, N. Y.; Charles Merritt, New York; G. A. Mills, Brooklyn; J. Frank Marriner, Ottawa, Ill.; W. H. Morgan, Nashville, Tenn; H. J. McKellops, St. Louis, Mo.; James McManus, Hartford, Conn.: J. T. McQuillen, Philadelphia; A. L. Northrop, New York; S. B. Palmer, Syracuse, New York; C. N. Pierce, Philadelphia; A. O. Rawls, Lexington, Ky.; F. H. Rehwinkel, Chillicothe, Ohio; Spencer Roberts, Philadelphia; T. C. Stellwagen, Philadelphia; H. L. Sage, Bridgeport, Conn.; L. D Shepard, Boston; C. Stoddard Smith, Springfield, Ill; D. D. Smith, Philadelphia; J. H. Smith, New Haven, Conn.; C. S. Stockton, Newark, N. J.; E. D. Swain, Chicago; J. B. Thompson, Newberry, S. C.; J. S. Thompson, Abbeville, S. C.; J. Taft, Cincinnati, Ohio; S. R. Thomas, Detroit; J. Wetherbee, Boston; A. J. Waide, Newtown, Iowa; W. T. Wallace, Kingsville, Ohio; Amos West, Philadelphia; M. H. Webb, Lancaster, Pa.; F. S. Whistler, Youngs town, Ohio.

A large number of others were present at the following sessions; (which continued until Friday noon, and ended with a very enjoyable excursion on the Steamer John A. Warner, up and down the Delaware River for some distance from Philadelphia, during which the visitors were very hospitably entertained by the Philadelphia Dentists by whom this excursion was gotten up,) among the number were Dr. Wm. H. Dwinelle, of New York; Drs. Arthur, Winder, Wilkerson, Waters, Ulrich and Uhler of Baltimore; Dr. Redman of Louisville, Ky.; Drs. Hoffman and Bland of North Carolina; Dr. T. J. Thomas of Madrid, Spain; Dr. Jno. G. Wayt, Richmond, Va.

The President, Dr. A. L. Northrop, of New York, called the meeting to order, with Dr. C. Stoddard Smith, ot Springfield, Illinois, Recording Secretary. After prayer by Dr. W. H. Morgan, of Nashville, Tennessee, the usual preliminary routine of business was observed.

Upon motion of Dr. L. D. Shephard, the rules were suspended to admit the introduction of a resolution permiting dentists not residents of the United States to occupy seats in the meeting and participate in the discussions, which was adopted.

Dr. A. H Brockway read a paper, treating the subject of "The health of the Dentist as influenced by the modern mode of practice." He considered the use of amalgam, non-cohesive gold and the combination of gold and tin, instead of adhesive gold, in many cases where the strength and physical ability of the dentist has been so largely taxed, as important conservators of health and energy. The enlightenment in the dental science by the invention of the mallet, the rubber saw, the burring engine and other appliances, has also proved a valuable help to health in the performance of numerous arduous operations.

Dr. Erastus Wilson, of Havana, Cuba, was introduced to the Association.

The suspension of the order of business was moved to hear the report of a committee appointed at the last meeting to inquire into the general health of dentists.

Dr. John Allen, of New York, then submitted the report which asserted that the peculiar nature of a heavy dental practice tends to overtax the system more than other professions. This is not only due to the close confinement to which dentists are subjected, but also to the intricate, long and trying operations which they are continually called upon to perform, and to their neglect of certain established universal and irrevocable laws of nature, distinguished among which is the baneful influence of stimulants and narcotics, to which they often resort for relief. A special recommendation was that operating rooms be arranged with a close regard to ventilation, and also that the office hours be lessened and more time devoted to recreation.

The Committee on Credentials presented a report of the names of delegates present from other Associations, which were as follows:

American Academy of Dental Science.—Prof. L. D. Shepard.

Chicago Dental Society—Dr. A. W. Harlan.

Susquehanna Dental Association—Dr. W. H. Hurtz.

Baltimore College of Dental Surgery—Prof. F. J. S. Gorgas, M. D.

New York Odontological Society—Drs. J. W. Clews, Benjamin Lord and William Jarvis, Jr.

Lake Erie Dental Association—Dr. G. B. McDonneld.

Odontographic Society—Drs. M. Long, C. A. Kingsbury, Alonzo Boice, William J. Potter and J. L. Elsenbrey.

North Carolina State Dental Association—Dr. W. H. Hoffman.

Kentucky State Dental Society—Dr. J. Hooper.

Philadelphia Dental College—Prof. Thos. C. Stellwagon.

First District Dental Society of the State of New York.—Drs. J. Bond Littig, and Charles Miller.

New York College of Dentistry—Dr. Wm. H. Allen.

Ohio State Dental Society—Dr. N. B. Acheson.

Pennsylvania Association of Dental Surgeons—Dr. Wm. H. Truman.

Alumni Association of the Penna. College of Dental Surgery—Dr. Henry W. Moore.

Iowa State Dental Society—Dr. A. J. Wald.

Penna. College of Dental Surgery—Prof. George T. Barker.

Penna. Association of Dental Surgeons—Drs. E. H. Neall, Amos Wirt and Spencer Roberts.

Connecticut Valley Dental Association—Drs. H. W. Strang and J. H. Smith.

Maryland Dental College—Prof. M. Whilldin Foster.

California State Dental Association—Dr. George McCamen.

Boston Dental College--Prof. I. J. Wetherbee.

New Jersey State Dental Society—Dr. Charles S. Stockton.

Illinois State Dental Society—Dr. C. S. Smith, M. S. Dean and J. N Crouse.

South Carolina State Dental Association—Dr. J. R. Thompson.

The following are the committees which reported at this session:

Dental Physiology.—Drs. J. H. McQuillen, S. W. Dennis, A. H. Brockway.

Pathology —Drs. J. Foster Flagg, L. D. Shepard, I. Knapp.

Chemistry.—Drs. S. B. Palmer, J. S. Cassidy, L. G. Noel.

Therapeutics.—Drs. W. H. Atkinson, C. A. Brackett, C. R. Butler,

Operative Dentistry.—Drs. Frank Abbott, A. W. Harlan, H. M. Reid.

Mechanical Dentistry.—Drs. W. N. Morrison, D. D. Smith, W. C. Barrett.

Etiology.—Drs. D. C. Hawxhurst, M. H. Webb, H. L. Sage.

Dental Literature.—Drs. M. S. Dean, E. D. Gaylord, G. C. Daboll.

Dental Education.—Drs. W. H. Morgan, J. N. Crouse, M. Wells.

Prize Essays.—Drs. F. H. Rehwinkel, Dr. G. W. Keely, H. J. McKellops.

Special Committee on the Health of Dentists.—Drs. John Allen, W. H. Morgan, W. E. Magill.

Executive Committee.—Drs. L. D. Shepard, M. H. Webb.

The discussion of the subject of the paper from the committeee on the General Health of Dentists, was opened by Dr. C. E. Kingsbury, who was followed by Dr. Barker, the latter maintaining in opposition that there was no single profession which was better adapted to prolong life and raise a higher type of manhood than dentistry. He thought that it was peculiarly adapted to the development of the mental and physical powers, whilst the class of patients that generally come under their care is such as tend to elevate and create a vital force within the dentist. What leads frequently to dyspepsia in the profession is the habit of bolting food at meals, which a constant professional practice sometimes seems to necessitate.

Without advocating the use of either liquor or tobacco, he maintained that alcoholic liquors should not be so greatly depreciated as Dr. Kingsbury had done, because they have their special uses in which they are priceless, especially in the treatment of consumption.

Dr. J. F. Flagg and Dr. Wetherbee both opposed Dr. Barker concerning the use of liquor, believing that they could and should be dispensed with in medical practice.

Dr. T. C. Stellwagen and others also spoke on the subject, objecting to practitioners administering intoxicating liquor in any other way than in very closely limited prescriptions. Other points relating to the habits and employment of dentists were also fully discussed.

The Committee on Credentials reported the names of Dr. W. McGrath, Pernambuco, Brazil; Dr. Nathaniel Emmons, Santiago-de-Chili; Dr. George Cunningham, London, and Dr. J. Carlos Gardiner, Madrid, as visiting dentists.

At the afternoon session an interesting paper submitted by Dr. J. H. McQuillen, as a report from the Committee on Physiology. The paper treated on the topic of "The Eruption of the Deciduous and Permanent Teeth," and stated that there were rare cases on record of children having been born with teeth, but usually the central incisors make their appearance between the sixth and eighth months of infancy, the lateral incisors between the seventh and ninth months, the first molars between the fourteenth and sixteenth months, the canines between the seventeenth and eighteenth months, and the second molars between the twenty-fourth and thirtieth months. About the period of the eruption of the first molar, a systematic disturbance occurs in most cases caused by the tooth, in its efforts to erupt through the gum, being compressed by the resistance of the gum upon the pulp, producing the consequences through the medium of the fifth pair of nerves. In explaining the method of eruption and disappearance of the deciduous teeth, and the mode of eruption of the permanent teeth, he called attention to the fact that his own comparisons with the recorded observations of Messrs. John Tomer and Edwin Saunders, of London, had manifested that eruption of the permanent teeth in America occurred considerably earlier than in England and France, thus seeming to indicate a precocity in our favor.

In speaking in relation to Dr. McQuillen's paper, Dr. Atkinson said that it had been established by authority, that twelve times the limit of the shedding of the central incisors is the normal length of life, therefore an early development in this respect was indicative of brevity of existence, while a later development seemed to predict longevity.

The discussion of the paper on the General Health of Dentists was continued in the afternoon and evening sessions.

### Second Day's Sessions.

The Association re-assembled in second-day's session at 10 A. M., Dr. A. L. Northrop, of New York, President, in the chair.

Dr. Frank Abbott, of New York, chairman of the Committee on Operative Dentistry, reported, giving a general retrospect of the science in the past century, and its advancement as indicated in the theories and practices of distinguished members of the profession from 1778 to to-day. In the earlier days the operating room was an exclusive and mysterious place, but with further enlightenment came a more liberal spirit, and in 1839 the Baltimore College of Dental Surgery was instituted, which was the first of a line of other colleges that are now distributed over the country. The mallet and pluggers were introduced in 1862, the rubber-dam in 1865, and the dental engine in 1870 or 1871. In conclusion, he said that, as practitioners of a specialty of a great and wonderfully useful profession, the necessity for a more positive prophylactic treatment of the organs committed to the dentist's charge is daily becoming more and more apparent.

A telegram of greeting was received from the American Dental Society of Europe, now in session at Paris, and a suitable reply was ordered to be sent.

Dr. A. W. Harlan, of Chicago, presented a supplemental report on Operative Dentistry, treating the subject of cohesive and non cohesive gold. The report, in closing, said —" Now, whilst I am willing to accord to those great intellects of the past great honor for what they did in their day and generation, for the upbuilding of the science of operative dentistry, I am not willing to accept the heritage left us and use it in modern dental practice, for the reason that something more is demanded of us.

As was remarked before, the teeth of the present generation are far inferior in structure to those operated on in the past. This fact alone makes it imperatively nscessary that we, in our operations, should as thoroughly as possible protect all surfaces of dentine and enamel where the nature of the decay demands it, and this we are unable to accomplish with non-cohesive gold. Again, I contend that in the successful use of cohesive gold the development of the highest grade of skill is attained, the nature of that form of gold itself compelling the operation to proceed step by step, until every part of the filling is completed ; thus, instead of being a hindrance, is a positive benefit, as it enables the operator to put to the best possible use whatever of artistic ability he may possess. That the result attained is far superior to that of any other, is unquestionably true ; for be it remembered, in the one case we have a perfectly coherent mass, and in the other the particles are only in apposition, and it matters not how well they were placed in position, no dependence can be placed on the strength of the filling itself, but, on the contrary, the walls and margins have to sustain all of the various requirements of mastication. We go a little further and say it is folly to suppose that we must make all of our fillings thoroughly dense, for that is not needed in all cases; but that it is more desirable for us to make them from a form of gold which possesses the element of cohesiveness, I am fully convinced."

Dr. W. N. Morrison, of St. Louis, upon the recommendation of the Executive Committee, gave various illustrations of cases where the operation of transplantation and replantation of teeth had been performed, in a volunteer paper.

Dr. J. Taft, of Cincinnati, also submitted a paper on the same subject, reciting forms of treatment as practiced in the past.

Dr. James Leslie, of Cincinnati, in speaking of the use of gold fillings, asserted that he was the real inventor of cohesive gold, in 1839, the first operation being performed at his suggestion by Dr. H. L. Klutt, of Louisville, Ky.

He believed one of the reasons to be ascribed to failures in gold filling was the use of the mallet. The principle of cohesion is impaired by the continued and repeated effects of the malleting, causing a hardening of the gold, and a consequent disintegration. He explained that a gold pellet or a leaf compressed and beaten into the cavity is only condensed by the driving out of the atmosphere from the interstices of the metal, and when the atoms are beaten together closer than the point where they are in contact, with the air entirely expelled, the disintegration is certain to take place.

Dr. Barker stated that it had been claimed that filmal resistance could not be overcome by gradual pressure, but only by shocks. He desired to know if this was a true theory, and if it was, he was going to retain the electric mallet. As to its use, he thought that all efficient dentists knew where the limitations in its employment were.

Dr. Wetherbee contended that cohesive foil can be completely and readily adapted to the walls of all cavities. He believed the use of the mallet was likely to bruise the dentine.

Dr. Shepard explained by black-board diagrams the philosophy of cohesion and non-cohesion in gold, showing why cohesive gold in rope form was unsuccessful in certain hands.

Dr. Flagg said that he had met with success in the use of layers of adhesive foil in many cavities, and he agreed with Dr. Wetherbee with regard to the dangers of the mallet.

Dr. Morgan, in speaking on this subject, said that in his investigation he had discovered that as far back as there is record of the use of gold, it has always been employed in the same identical forms in which it is now used.

Dr. Stellwagen said that what makes dentistry a profession is the power to judge where it is advisable to employ cohesive and where it is best to employ non-cohesive gold. Neither form could be used exclusively in general practice, but the specific usefulness of both ought to be universally recognized.

Dr. Hunt said that it was recognized that fillings of both kinds of foil were often successful, but what ought to be learned by the profession is the exact action of the gold in its adhesion in the tooth. Hydraulic pressure can be made to burst the strongest vessel; lead poured into an iron cup and hammered, the cup will burst; what then will be the effect of malleting upon gold placed in the cavity of a tooth?

Dr. Barker said that the usefulness of cohesive and non-cohesive gold depended, not so much upon the superior qualities of the one or the other, as it does upon the skill of the operator. Thus it was a mistake for those who were less skillful to attribute their inability to perform a successful operation to defects in the form of gold which they employ, while the true causes lie in their own incompetence.

He had often carefully examined both cohesive and non-cohesive gold by microscopic analysis, and had never been able to detect the difference.

At 1 P. M. the meeting adjourned until 2-30 P. M., when, upon reassembling, Dr. Harker continued his explanation of a means of treatment in replantation that had been interrupted.

Dr. Stellwagon exhibited a rooster, in the comb of which had been transplanted a bicuspid. In relation to the permanency of transplantation, he said that he believed there were instances of teeth lasting nineteen or twenty years. He spoke particularly of the necessity for extreme caution in the selection of such teeth for transplantation as will not transfer any hereditary diseases.

Dr. Taft recommended the transplantation in many cases of alveolar abscesses where there is chronic inflammation of the tissue around the tooth resisting all ordinary means to restore a healthy condition. He had employed the treatment in such cases himself, taking out the tooth, cleansing the socket of all debris and replacing the tooth again. It was best applicable, he thought, to teeth of a single roof. In cases of neuralgia it was also recommended as a useful process. Care in every case should be taken with the transplanted tooth that it may be kept in a perfect condition ; antiseptics should be used to prevent decomposition, and the patient should be placed in a nutritive condition of system and protected from the many outside influences.

Dr. Atkinson briefly considered the sympathetic energy necessary to cause the transplanted tooth to unite with the tissues of the gum. He contended that it was an assumption for any dentist to talk about influencing constitutional conditions so as to produce consequences necessary to cause the union.

Dr. Palmer, in speaking of a new method for the preparation of gold foil, said that it should be cut into sheets, and placed in the books by the beater without the preliminary heating, the operator using it just as it comes from the beater, doing the heating himself before placing it in the cavity. The repeated heating of the foil by the beater reduces the cohesive qualities of the metal.

Dr. Buckingham, from the committee appointed to prepare suitable action on the death of Professor Wildman, submitted a series of resolutions eulogistic to the character of the deceased, which were adopted, after remarks by Dr. Buckingham concerning Dr. Wildman's valuable labors in the profession.

The Association then adjourned until evening.

In the evening, the Association met in the hall of the Philadelphia College of Dental Surgery, North-West Cor. of Tenth and Arch Streets, when the session was opened by Dr. McQuillen, who continued the discussion of the papers on operative dentistry.

Dr. Butler said that, under some conditions, teeth were better filled with other metals than gold, and he believed that more attention should be given to this variety of conditions. He spoke especially in favor of tin for use in filling children's teeth in certain forms of decay.

The subject, after much further debate, was passed, and a report from the Committee on Dental Chemistry was read by Dr. Palmer, of Chicago, quoting largely from the dental literature of the year concerning the progress made in this particular branch.

The discussion of this paper was pending when the Association adjourned until this morning.

### Third Day's Sessions.

The Association re-assembled in third day's session at 10 A. M., Dr. A. L. Northrop, of New York, in the chair.

The Treasurer, Dr. W. H. Goddard, of Louisville, Ky., submitted his report, showing that the receipts of the year, including a balance of $189.75 from the preceeding year, amounted to $839.75, while the expenditures were $664.75, thus leaving a balance of $175 in the treasury.

Dr. L. D. Shepard, of Boston, read a volunteer paper written by Dr. J. S. Cassidy, of Cincinnati, on "Sleep versus Anæsthesia." Discussing the chemical changes involved in sleep and anæsthesia the paper stated that all such physiological manifestations are due to the evolution of carbonic acid gas, differing in force and degree. The comparative effects and safety of different anæsthetics depend upon the rapidity or slowness of the exhalation of the said gas.

Dr. J. Taft, of Cincinnati, offered a paper on "Solution." The author in this defined solution to be the transfer of solid substances into the fluid form, involving the atomic division of the substance dissolved. This is accomplished by suspending or overcoming the cohesive atomic affinity by the influence of the solvent. Many circumstances modify solution, prominent among which are the character of the menstrum, the character of the substance to be dissolved, heat, light, electricity, pressure, and the presence of a third substance. In dissolving many substances heat is evolved, in others cold is expressed. Quite a number of experiments were referred to illustrating these principles. Solution, it was stated, is more than a mere suspension of a fluid. Solution in vegetable and animal processes is involved in much obscurity, but nevertheless is worthy of careful consideration, involved as it is in and connected with all the work of growth and development.

Professor D. D. Smith, of Philadelphia, asserted that the paper of Dr. Taft had nothing to do with dentistry, and he hoped that the views therein expressed would not meet with a ready acceptance from the members of the Association. In relation to his and others, experience at Boston, in operating with gold filling, he said that in teeth so filled the cervical walls lasted longer than the grinding parts, but in Philadelphia the result seemed to be reversed, and to discover the cause it was necessary to consult chemistry. He advocated amalgam as useful in the preservation of chalky teeth and preferable to gold. This was because the galvanic current created by the presence of amalgam and dentine was not as strong as that between dentine and gold.

Dr. Wetherbee said that he thought that the secretions of the mouth determined the action upon the filled teeth as much as did the galvanic current. In amalgam are ingredients more susceptible of galvanic action than in gold.

Dr. Barker spoke to a question of privilege. He said exception had been taken to the views advanced by him on Wednesday, when the subject of replantation was under consideration. One gentleman, ignoring all physiological and pathological facts, had claimed that after the extraction of a tooth, it could be returned to its socket and that it was possible for union of the divided pulp to occur, leaving the tooth alive, as it was formerly. To this possibility or even probability he (Dr. Barker) desired to enter his protest, and considered that, while under some circumstances replantation of teeth is advisable and practical, it is not possible to preserve the pulp alive, and if retained will produce disease by its presence.

Dr. Clowes said that the success of the filling operations depended not so much on the material used, as the care taken to interrupt the electrical current.

Dr. G. A. Mills thought that cleanliness in the preparation of the cavity was a consideration of paramount importance. Close attention should always be given to this matter, and its necessity duly enforced upon patients.

Dr. Cunningham, of England, upon being invited, spoke on the subject of "Elctro-Chemistry of the Dental Tissues," explaining certain experiments made by himself.

Dr. J. F. Flagg spoke against the extraction of teeth in many cases where they could be preserved by filling. He also spoke in strong advocacy of plastic material in the place of gold.

Dr. Marshall Webb maintained an exactly opposite position on the subject.

Dr. Shepard read a report from the committee on Dental Therapeutics, written by Dr. C. A. Brackett, of Newport, Rhode Island.

Dr. W. H. Atkinson, of New York, also submitted a report from the same committee.

The Association then took a recess until 2.30 P. M.

Upon re-assembling, the discussion of the subject of therapeutics was resumed by Dr. Barker, who called attention to the usefulness of salicylic acid as an antiseptic; not so valuable as creosote or carbolic acid, but yet very applicable to certain cases. He also directed attention to a statement in Dr. Atkinson's paper, that no change could be expected in the condition of teeth after adult age by the administration of materials which could, by absorption into the system, be appropriated and thereby improve the dental structures. He took exception to this position, and cited cases where remarkable improvement had occurred from the use of the syrup of lacto-phosphate of lime. This remedy, in connection with good food and the observance of hygienic laws, would be followed by favorable results, particularly in cases of debility of nursing mothers, when a double benefit is derived, not only for the mother, but for the child as well.

Dr. Dixon fully coincided with Dr. Barker in regard to the changes in the teeth after adult life. He was convinced that these changes take place at all periods of life, and sometimes with startling rapidity. No dentist is justified in predicting that a person's teeth will last for a lifetime or even for a single year.

Dr. Hunt said that in the use of therapeutical agents they should be brought to that condition in which they nearly approach the products of nature.

Dr. John Allen said that he thought that therapeutic agents originating in nature's own laboratory would assimilate to the proper organisms of the body better than any that could be produced by artificial means.

Dr. W. N. Morrison, of St. Louis, read a report from the Committee on Mechanical Dentistry, presenting to the notice of the Association a patented porcelain pinless tooth.

Dr. D. D. Smith, of Philadelphia, read a paper written by Dr. W. C. Barrett, of Warsaw, N. Y., on the subject of rubber and celluloid fillings as adapted to the necessities of those whose circumstances will not permit the use of gold.

Dr. Smith also made a verbal report, reviewing the subject of mechanical dentistry to indicate exactly its present state of advancement.

Dr. D. C. Hauxhurst, of Michigan, presented a report from the Committee on Etiology. This paper took the ground that the jaws of nations are lessening in size and changing their shape, as the nation advances in civilization. The causes ascribed to these changes were the use in masticating injurious food, hereditary transmission from parents and the intermarriage of different nationalities. He also directed attention to the retrograde of the teeth from the same causes.

Dr. Marshall Webb, of Lancaster, Pa., read a paper giving a clear exposition of the pathological changes which take place at the roots of teeth, the sequence of dental disease.

Another paper on the same subject was read by Dr. H. L. Sage, of Bridgeport, Conn., which referred to the material influences which modify the teeth of infants, and adverted to the effects produced inducing disturbance of health during dentation.

Mention was made of the influence of the blood, when impoverished, in producing disease, and also of the universal presence of decay on the teeth of children from these causes. He spoke of the nervous diseases of parents, which predispose their children to weakened constitution, and, as a consequence, to dental disease.

The Association then adjourned until evening.

In the evening it was determined that the next meeting be held in Chicago, that city receiving a majority over Deer Park, Md., situated on the Baltimore and Ohio Railroad.

The ballot for officers resulted in the election of the following:

President, Dr. G. W. Keely, of Oxford, Ohio ; 1st Vice-President, Dr. C. N. Pierce, of Philadelphia ; 2nd Vice-President, Dr. Cordon Palmer, of New York ; Corresponding Secretary, Dr. J. H. McQuillen, of Philadelphia ; Recording Secretary, Dr. C. Stoddard Smith, of Springfield, Ill ; Treasurer, Dr. W. H. Goddard, of Louisville, Ky.

The Association then adjourned to meet again Friday morning.

Upon the adjournment of the Association, the members were invited by the Philadelphia dentists to enjoy an encursion on the Delaware River, the steamer John A. Warner leaving Chestnut Street wharf at 2 P. M. Friday, and after proceeding some distance up the river, the steamer to return to the city, stopping at the wharf at 3-50 P. M. on her way down the river.

### Fourth Day's Session.

The Association re-assembled at 9 o'clock Friday morning, the President, Dr. A. L. Northrop, in the chair. The Chairman of the Committee on Dental Literature, Dr. M. S. Dean, read a very voluminous report on this subject, referring especially to the different dental journals, and advising the members of the Association to support them by subscriptions and articles. He also spoke in flattering terms of the *British Dental Journal*, and recommended its being added to the list of those of our own country by all practitioners who could afford such an expense.

The Chairman of the Committee on Dental Education, Dr. W. H. Morgan, then presented the report on this subject, but owing to the want of time and some confusion, incident to the end of the sessions, no discussion followed on any of the reports presented at Friday's session. Dr. W. H. Dwinelle, of New York, also read a very excellent paper in Dental Literature, in which he referred to the efforts in behalf of the science, by the pioneers of the profession, and the results of their labors as manifested at the present time, especially those of Drs. Hayden, Chapin A. Harris, Thos. E. Bond, and others; the establishment of the AMERICAN JOURNAL OF DENTAL SCIENCE, and of the Baltimore College of Dental Surgery under a charter granted by the Legislature of the State of Maryland.

The chairman of the committee on Dental Appliances, Dr. G. L. Field, unsuccessfully endeavored to obtain a hearing for the report on this subject, very much to the regret of a number of the members present. It was determined, however, to allow the chairman an opportunity if possible, to make this report on board the Steamer during the excursion. The President elect, Dr. G. W. Keely, of Oxford, Ohio, was then installed, and the committees for the ensuing year announced as follows:

*Physiology.*—Drs. M. S. Dean, R. B. Winder, J. N. Farrer.

*Pathology and Surgery.*—Drs. I. Knapp, J. Taft, F. H. Rehwinkel.

*Histology and Microscopy.*—Drs. C. N. Pierce, E. D. Swain, H. C. Longnicker.

*Chemistry.*—Drs. S. B. Palmer, J. S. Cassidy, C. J. Essig.

*Therapeutics.*—Dr. C. A. Brackett, C. R. Butler, F. M. Odell.

*Operative Dentistry.*—Drs. G. H. Cushing, A. W. Harlan, F. J. S. Gorgas.

*Mechanical Dentistry.*—Drs. W. N. Morrison, F. A. Hunter, M. W. Foster.

*Etiology.*—Drs. D. C. Hawkhurst, M. H. Webb, H. L. Sage.

*Literature.*—Drs. W. A. Bronson, A. L. Northrop, E. A. Bogue.

*Education.*—Drs. L. Jack, J. N. Crouse, T. Fillebrown.

*Prize Essays.*—Drs. W. H. Morgan, B. Lord, L. D. Shepard.

At 1 P. M. the Association adjourned to meet in Chicago, on the first Tuesday in August, 1877.

